# Smooth Muscle Endothelin B Receptors Regulate Blood Pressure but Not Vascular Function or Neointimal Remodeling

**DOI:** 10.1161/HYPERTENSIONAHA.115.07031

**Published:** 2017-01-11

**Authors:** Eileen Miller, Alicja Czopek, Karolina M. Duthie, Nicholas S. Kirkby, Elisabeth E. Fransen van de Putte, Sibylle Christen, Robert A. Kimmitt, Rebecca Moorhouse, Raphael F.P. Castellan, Yuri V. Kotelevtsev, Rhoda E. Kuc, Anthony P. Davenport, Neeraj Dhaun, David J. Webb, Patrick W.F. Hadoke

**Affiliations:** From the University/BHF Centre for Cardiovascular Science, University of Edinburgh, United Kingdom (E.M., A.C., K.M.D., N.S.K., E.E.F.v.d.P., R.A.K., R.M., R.F.P.C., N.D., D.J.W., P.W.F.H.); University of Basel, Switzerland (S.C.); Centre for Functional Genomics, Skolkovo Institute of Science and Technology, Russian Federation (Y.V.K.); and Division of Experimental Medicine and Immunotherapeutics, Addenbrooke’s Hospital, Cambridge, United Kingdom (R.E.K., A.P.D.).

**Keywords:** autoradiography, endothelin-1, hypertension, neointima, vasoconstriction

## Abstract

The role of smooth muscle endothelin_B_ (ET_B_) receptors in regulating vascular function, blood pressure (BP), and neointimal remodeling has not been established. Selective knockout mice were generated to address the hypothesis that loss of smooth muscle ET_B_ receptors would reduce BP, alter vascular contractility, and inhibit neointimal remodeling. ET_B_ receptors were selectively deleted from smooth muscle by crossing floxed ET_B_ mice with those expressing cre-recombinase controlled by the transgelin promoter. Functional consequences of ET_B_ deletion were assessed using myography. BP was measured by telemetry, and neointimal lesion formation induced by femoral artery injury. Lesion size and composition (day 28) were analyzed using optical projection tomography, histology, and immunohistochemistry. Selective deletion of ET_B_ was confirmed by genotyping, autoradiography, polymerase chain reaction, and immunohistochemistry. ET_B_-mediated contraction was reduced in trachea, but abolished from mesenteric veins, of knockout mice. Induction of ET_B_-mediated contraction in mesenteric arteries was also abolished in these mice. Femoral artery function was unaltered, and baseline BP modestly elevated in smooth muscle ET_B_ knockout compared with controls (+4.2±0.2 mm Hg; *P*<0.0001), but salt-induced and ET_B_ blockade–mediated hypertension were unaltered. Circulating endothelin-1 was not altered in knockout mice. ET_B_-mediated contraction was not induced in femoral arteries by incubation in culture medium or lesion formation, and lesion size was not altered in smooth muscle ET_B_ knockout mice. In the absence of other pathology, ET_B_ receptors in vascular smooth muscle make a small but significant contribution to ET_B_-dependent regulation of BP. These ET_B_ receptors have no effect on vascular contraction or neointimal remodeling.

## Abstract 2

Supplemental Digital Content is available in the text.

Endothelin-1 (ET-1), released by vascular endothelial cell (EC) and inner medullary collecting duct cells (and other cells under pathological conditions), stimulates endothelin_A_ (ET_A_) and endothelin_B_ (ET_B_) receptor subtypes.^1,2^ ET_A_ are present on vascular smooth muscle cells (VSMCs), predominantly mediating contraction^3^ and regulating blood pressure (BP).^4^ They also influence mitogenesis,^5^ generation of reactive oxygen species, and adhesion molecule expression.^6,7^ ET_A_ receptors on leucocytes mediate cytokine release and cellular chemotaxis.^8^ Many of these processes contribute to vascular remodeling, and ET-1 clearly drives arterial lesion formation (including neointimal proliferation after injury).^7^ This can be inhibited by selective ET_A_ antagonism.^9,10^

Regulation of arterial function, BP, and arterial lesion formation by ET_B_ receptors is likely to be more complex because they are expressed in EC, VSMC, and the kidney where they mediate physiologically antagonistic responses. ECET_B_ receptors mediate production of vasodilator, antiproliferative, and anti-inflammatory molecules (eg, nitric oxide [NO])^11,12^; clearance of ET-1 from the circulation^13,14^; and regrowth of damaged EC.^15^ VSMC ET_B_ can mediate vascular contraction, similar to the ET_A_ subtype,^16^ and may compensate for ET_A_ receptor dysfunction.^17^ ET_B_ upregulation in VSMC may mediate vasoconstriction and proliferation in cardiovascular disease.^18,19^

ET_B_-dependent regulation of BP is demonstrated by the sustained hypertension caused by ET_B_ receptor antagonism in mice.^20^ The importance of receptor distribution in this response is indicated by increased BP after deletion of ET_B_ receptors in the renal collecting duct^21^ but not after deletion of ECET_B_.^22^ The influence of VSMC ET_B_ on BP has not been established but, given their potential to mediate vasoconstriction, deletion or antagonism of VSMC ET_B_ would be predicted to reduce BP.

Despite the influence of ET-1 in vascular remodeling,^23^ the role of ET_B_ is less clear. ET_B_ activation in EC (NO release) and kidney (reduced BP) would be predicted to inhibit arterial remodeling, thus favoring selective ET_A_ antagonism for reducing neointimal proliferation.^9^ Certainly, global deletion of ET_B_ receptors increases vascular lesion size.^10,24^ However, selective ECET_B_ deletion did not influence lesion formation, suggesting that the protective role was mediated by ET_B_ receptors in other tissues.^9^ If ET_B_ receptors in VSMC contribute to lesion formation, mixed ET_A/B_ antagonists might have advantages over ET_A_ selective compounds, although recent investigations^9,10,24^ favor the latter.

We generated novel smooth muscle ET_B_ receptor knockout (SMET_B_ KO) mice to address the hypothesis that loss of these receptors would impair arterial contraction, lower BP, and reduce neointimal lesion formation in response to vascular injury.

## Methods

Mice with VSMC-selective ET_B_ receptor deletion were generated by crossing homozygous floxed ET_B_ mice with SM22cre transgenic mice, which express cre-recombinase in the heart and smooth muscle, (then backcrossed to a C57Bl/6J background for 4–6 generations), as described for ECET_B_ KO.^22^ Controls were Cre-negative littermates (ET_B_^f/f^). Genotyping was performed using ear clips.^22,25^ Wild-type (WT) C57Bl/6J mice were from Charles River (United Kingdom). Mice were housed according to the UK Home Office recommendations (22°C; 12-hour light/dark cycles) with free access to water and chow. Procedures were performed under the provisions of the Animals Scientific Procedures Act (1986) and approved by the local Ethics Committee.

Selective SMET_B_ deletion was demonstrated in organs and in isolated aortic smooth muscle cells (SMCs) using polymerase chain reaction, autoradiography,^14,26^ immunohistochemistry,^27^ and functional (myographic) investigation of isolated trachea, arteries, and veins.^28,29^

The impact of SMET_B_ KO on BP was assessed using radiotelemetry^22^ in conscious, unrestrained male SMET_B_ KO mice and age-matched controls (n=8 per group), fed on chow (7 days), high (7.6%) salt diet (7 days), then high salt plus ET_B_ antagonist (SB192621; 30 mg^−1^ kg^−1^ day^−1^ in drinking water, 7 days). ET-1 concentrations in plasma from WT C57Bl/6J, controls, and SMET_B_ KO were measured after exposure to chow or to high salt diet plus ET_B_ antagonist, by ELISA (Endothelin-1 Quantikine ELISA kit; R&D Systems, Oxford, United Kingdom).

Intraluminal (left) or nondenuding (right) femoral artery injury was achieved by insertion of an angioplasty guidewire or ligation, respectively, as described.^9^ After 28 days, arteries were retrieved (after perfusion fixation) and analyzed using optical projection tomography, histology, and immunohistochemistry.^9,30^

### Statistics

Results are mean±SEM, for n mice. Group sizes were chosen to detect 5%, 20%, and 20% differences in BP (n=7), lesion size (n=7), and maximum responses to vasoactive agents (n=6) with >90% power. Investigations were performed by operators blinded to treatment. Components of lesions were expressed as a percentage of the neointimal area. Analyses were performed with GraphPad Prism using Student *t* test, 1-way or 2-way ANOVA with a Tukey post hoc test, as indicated. Significance was assumed for *P*<0.05.

Detailed methods are in the online-only Data Supplement.

## Results

### Identification of SMET_B_ KO

**Figure 1.**
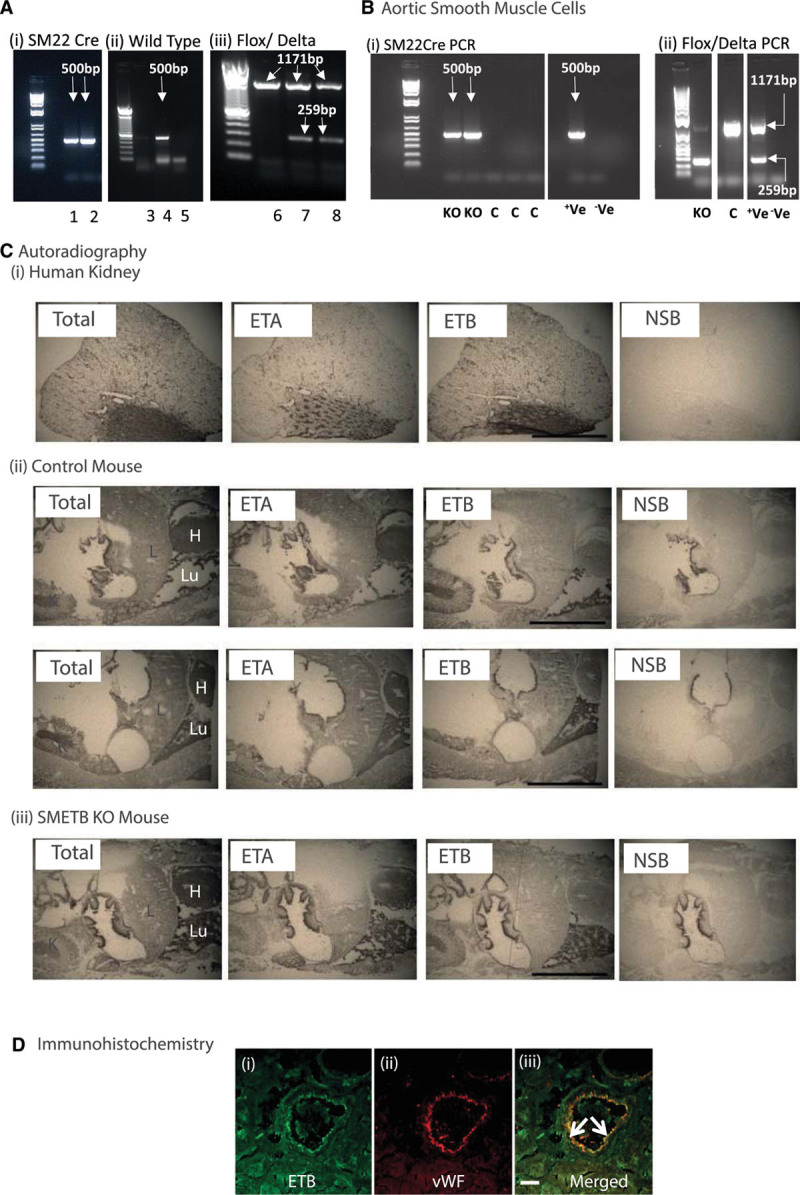
Selective endothelin_B_ (ET_B_) receptor deletion from smooth muscle. **A**, Mice were genotyped for (**i**) SM22cre (band at 500 bp), (**ii**) wild-type (band a 500 bp), and (**iii**) flox (band at 1171 bp)/delta (band at 259 bp) alleles in ear clip DNA. (**i**) Samples 1 and 2 are cre-positive, (**ii**) sample 4 is positive for the wild-type allele; samples 3 and 5 are not, (**iii**) samples 7 and 8 are positive for both the flox and the delta band; sample 6 has only the flox band. **B**, Polymerase chain reaction (PCR) for cre and flox/delta bands in murine aortic smooth muscle cells isolated from smooth muscle ET_B_ receptor knockout (SMET_B_ KO) and control (C) mice. Control mice lacked cre and delta alleles, whereas SMET_B_ KO expressed all 3. Standard DNA ladders have band sizes 1500–100 bp. **C**, Autoradiography showing maintained ET_B_ ligand binding in SMET_B_ KO lung and kidney (representative of n=3 mice/genotype). **D**, Confocal images of a coronary artery from an SMET_B_ KO mouse stained for (**i**) ET_B_ receptor (green) or (**ii**) the endothelial cell marker von Willebrand factor (vWF; red). Merged images (**iii**) show clear colocalization of ET_B_ with the endothelium (arrows). There is no ET_B_ staining in medial smooth muscle. Scale bar=50 µm. +Ve, positive control; –Ve, negative control; ET_A_ indicates endothelin_A_; H, heart; K, kidney; L, liver; Lu, lung; and NSB, nonspecific binding.

Genotyping for SM22cre, WT, and delta band alleles (Figure 1A) identified SMET_B_ KO (positive for SM22cre, floxed, and delta band and negative for WT allele) and controls (SMET_B_^f/f^ cre-negative littermates; negative for WT allele, positive for floxed allele, and negative for SM22cre and delta band). SMC isolated from the aorta of SMET_B_ KO mice expressed the cre, delta, and flox bands, whereas controls did not express the cre and the delta bands (Figure 1B).

Autoradiography (Figure 1C) identified ET_B_ receptors in the gut lining, lung, and kidney. This signal was not diminished after SMET_B_ deletion. ET_B_ expression (real-time polymerase chain reaction) was not altered in the colon, heart, or gastrocnemius muscle of SMET_B_ KO mice (Figure S1 in the online-only Data Supplement). Confocal imaging of immunofluorescence (Figure 1D) clearly showed ET_B_ receptors localizing to the endothelium (von Willebrand factor positive) in SMET_B_ KO coronary artery. ET_B_ staining in medial SM remained at background levels. This confirms maintained ET_B_ receptor expression in the endothelium of SMET_B_ KO mice.

### Functional Confirmation of SMET_B_ KO

SMET_B_ KO mice were healthy with normal body and organ weights (Table S1).

**Figure 2.**
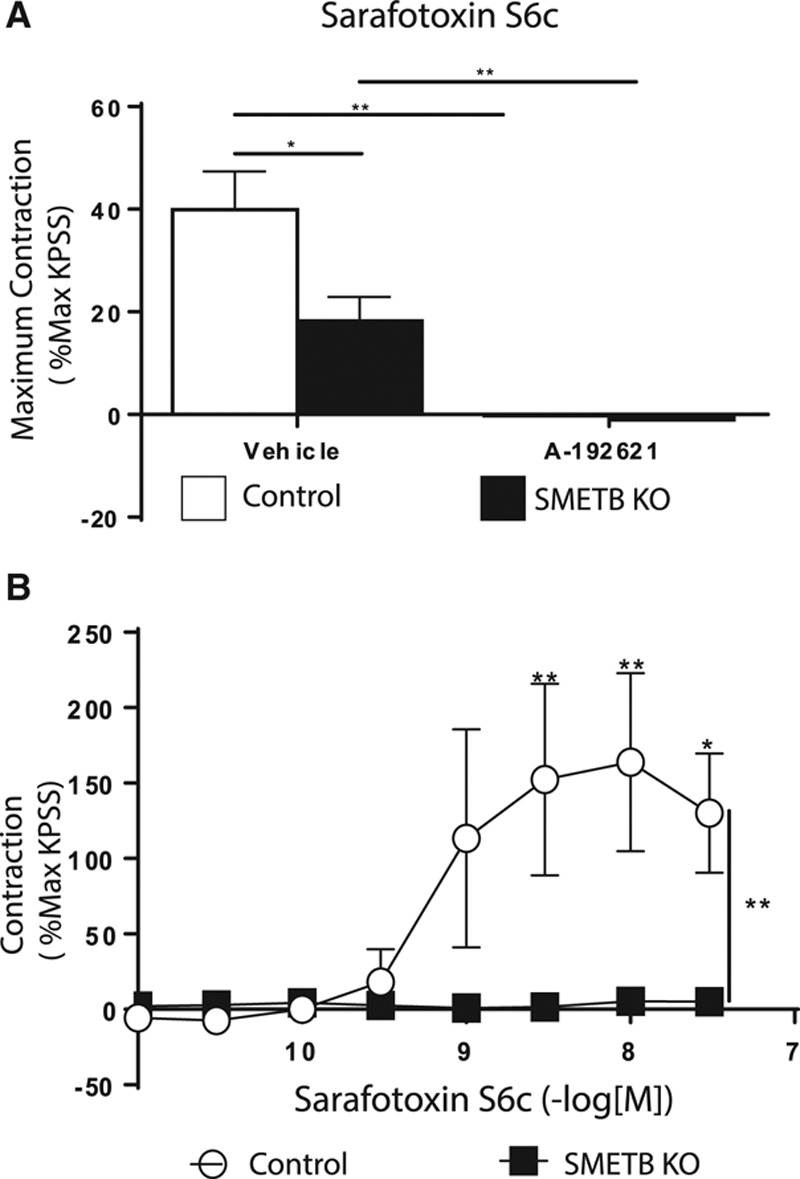
Functional consequences of selective endothelin_B_ (ET_B_) deletion from smooth muscle (SM). **A**, Sarafotoxin S6c (S6c)-induced contraction of isolated trachea was abolished by ET_B_ receptor antagonism (A192621; 100 nmol/L) but only reduced by selective smooth muscle ET_B_ receptor (SMET_B_) deletion (residual contraction was blocked by A192621). Columns are mean±SEM (n=4). *P<0.02, **P<0.005. **B**, S6c-induced contraction in murine mesenteric veins was abolished by SMET_B_ deletion. Symbols represent mean±SEM (n=4). *P<0.05, **P<0.01. KO indicates knockout; and KPSS, potassium physiological salt solution.

Sarafotoxin S6c (S6c)–mediated contraction in tracheas (which express ET_B_ receptors on SM)^22^ from controls was abolished by incubation with the selective ET_B_ antagonist A192621 (Figure 2A).^22^ In SMET_B_ KO mice, S6c-mediated contraction was reduced (≈30%), but not abolished. The residual contraction was blocked by ET_B_ antagonism. S6c-mediated contraction of mesenteric veins was abolished by selective deletion of SMET_B_ (Figure 2B).

### SMET_B_ KO and BP

**Figure 3.**
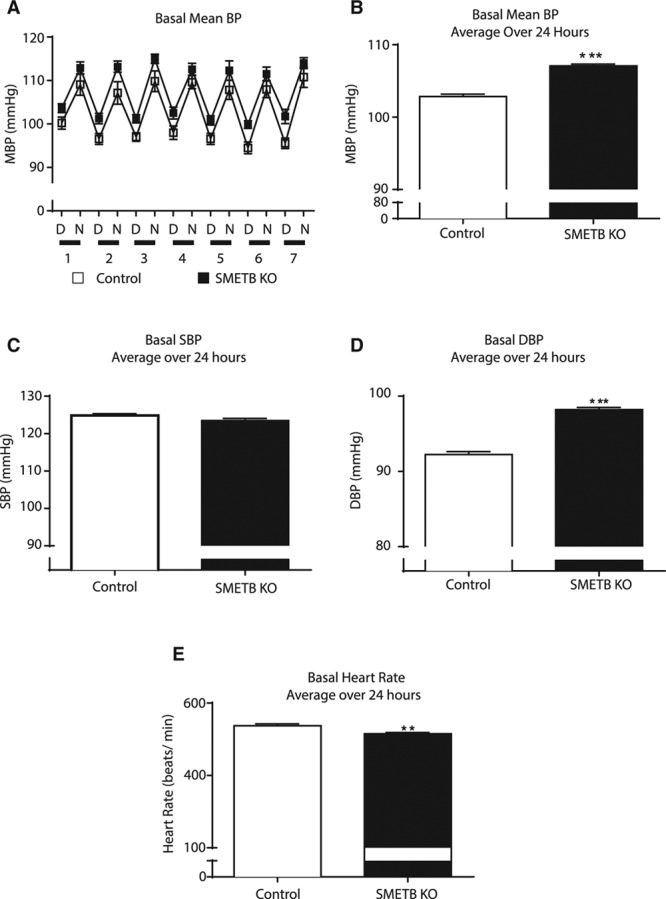
Selective deletion of endothelin_B_ (ET_B_) receptors from smooth muscle increases baseline blood pressure (BP). **A**, BP, assessed in conscious, unrestrained male smooth muscle ET_B_ receptor knockout (SMET_B_ KO) mice and controls (n=8 per group) using radiotelemetry, demonstrated a clear diurnal rhythm. Mean blood pressure (MBP) in SMET_B_ KO (filled symbols) mice was consistently higher than controls (open symbols). **B**, Data averaged over 24 h confirmed elevated MBP in SMET_B_ KO, with no difference in (**C**) systolic blood pressure (SBP) but (**D**) elevated diastolic blood pressure (DBP). **E**, Increased MBP was accompanied by reduced heart rate. Data are mean±SEM (n=8 per group). **P<0.005, ***P<0.0001. D indicates day; and N, night.

**Figure 4.**
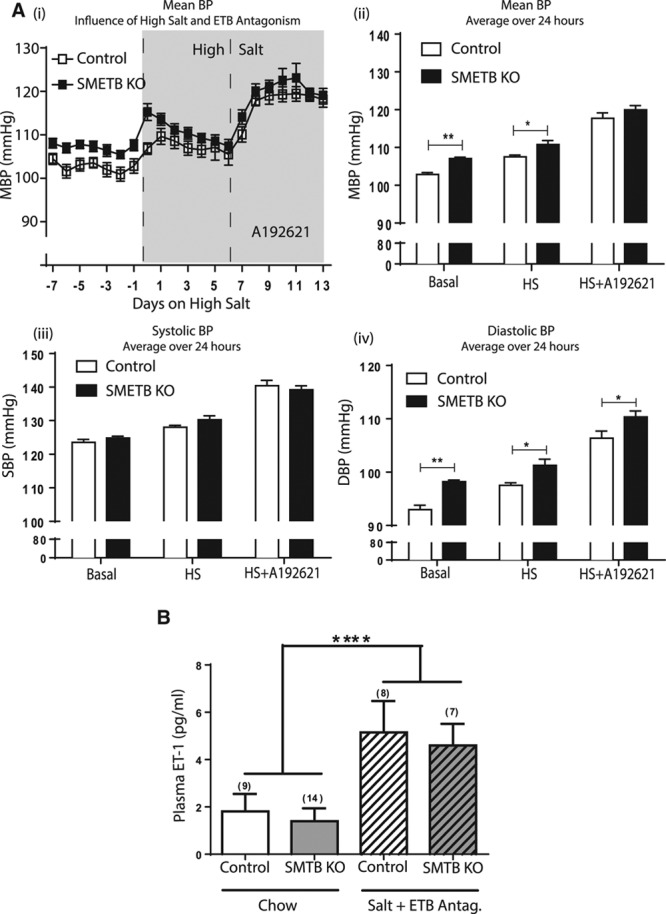
Selective deletion of endothelin_B_ (ET_B_) receptors from smooth muscle does not alter blood pressure (BP) responses. **A**, BP, assessed in conscious, unrestrained male smooth muscle ET_B_ receptor knockout (SMET_B_ KO) mice and controls (n=8 per group) using radiotelemetry (**i**) was elevated by high salt diet (HS; 7 d) and by ET_B_ antagonism (A192621; 30 mg^−1^ kg^−1^ d^−1^; 7 d) in both groups. (**ii**) Comparison of BP (averaged over 24 h) demonstrates the elevation in mean blood pressure (MBP) in response to high salt diet and high salt diet plus A192621. (**iii**) There was no difference in systolic blood pressure (SBP) in control compared with SMET_B_ KO mice but (**iv**) diastolic blood pressure (DBP) was higher in SMET_B_ KO for all treatment groups. **B**, Plasma endothelin-1 (ET-1) concentrations were similar in SMET_B_ KO and controls and consistent with wild-type C57Bl/6J mice (1.14±0.08 pg/mL; n=6). ET-1 concentrations were elevated in control and SMET_B_ KO mice after exposure to a high salt diet plus A192621. Data (mean±SEM) were analyzed using 2-way ANOVA with Tukey or Bonferroni post hoc test, as appropriate. **A**, *P<0.05, **P<0.01 compared with controls. **B**, ****P<0.00001 (effect of diet).

Control and SMET_B_ KO mice demonstrated a clear diurnal rhythm in BP (Figure 3A). Mean 24-hour BP was higher in SMET_B_ KO mice than in controls (107.1±0.3 versus 102.8±0.5 mm Hg; n=7; *P*<0.0001; Figure 3B). Systolic BP was not different between groups (123.5±0.6 versus 124.8±0.5 mm Hg; *P*=0.09; Figure 3C), but SMET_B_ KO mice had an increased diastolic BP (98.2±0.3 versus 92.2±0.4 mm Hg; *P*<0.0001; Figure 3D). BP elevation occurred despite reduced heart rate (515±3 versus 538±5 bpm; *P*=0.004; Figure 3E). High salt increased BP in controls with a further increase induced by ET_B_ antagonism (Figure 4A). These responses were similar in SMET_B_ KO.

### SMET_B_ KO and Circulating ET-1

Plasma ET-1 concentrations were similar in SMET_B_ KO and control mice (Figure 4B) and consistent with levels in WT C57Bl/6J (1.14±0.08; n=6). The combination of high salt diet and ET_B_ antagonism increased plasma ET-1 to a similar extent in control-type and SMET_B_ KO mice (Figure 4C).

### SMET_B_ KO and Neointimal Remodeling

**Figure 5.**
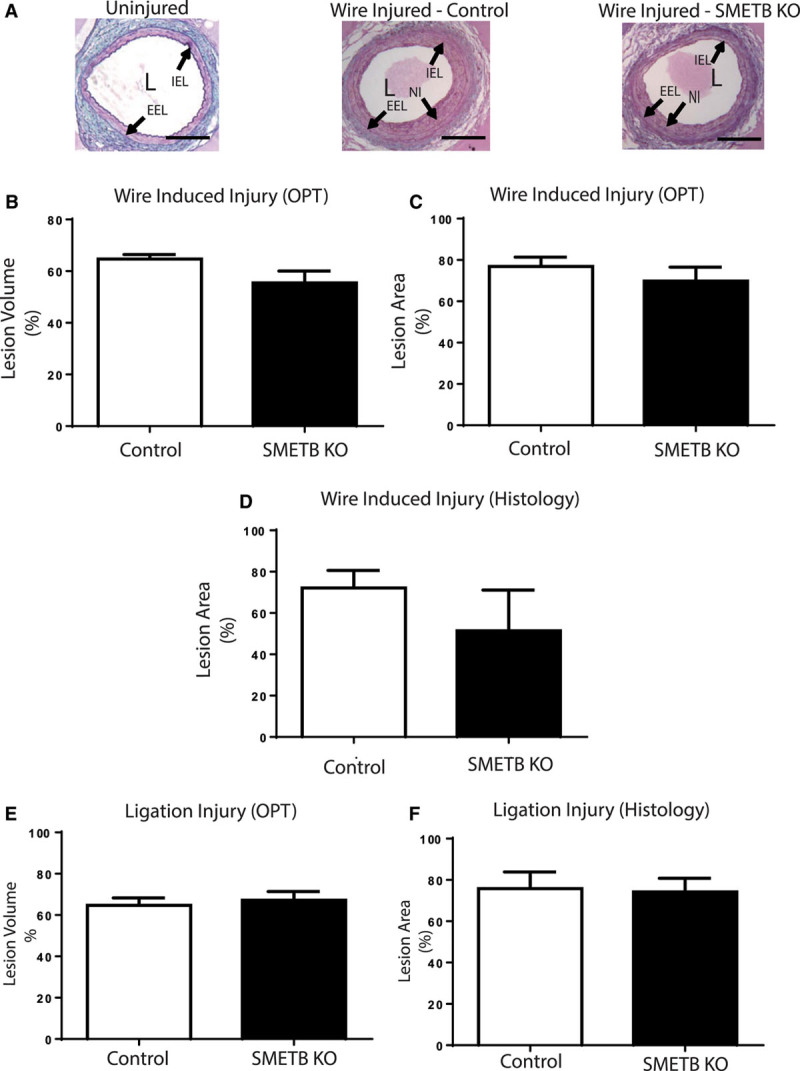
Selective smooth muscle endothelin_B_ (SMET_B_) deletion does not alter neointimal lesion formation. **A**, Wire injury–induced lesion formation in femoral arteries from control and SMET_B_ knockout (KO) mice. Neointimal lesion volume (**B**) and maximal cross-sectional area (**C**) were similar in control and SMET_B_ KO mice when measured by optical projection tomography. Similar results were obtained when maximal cross-sectional area was measured histologically (**D**). Volume (**E**) and maximal cross-sectional area (**F**) of lesions induced by ligation were similar in control and SMET_B_ KO mice (optical projection tomography [OPT]). Data are mean±SEM (n=7). EEL indicates external elastic lamina; IEL, internal elastic lamina; L, lumen; and NI, neointima.

Wire injury of the left femoral artery generated neointimal lesions (Figure 5A).^9^ Optical projection tomography demonstrated that SMET_B_ KO altered neither the lesion volume (Figure 5B) nor cross-sectional narrowing (Figure 5C). Histological analysis showed a trend toward reduced cross-sectional narrowing in SMET_B_ KO (Figure 5D). Ligation of the right femoral artery generated lesions^9^ with similar volume (Figure 5E) and maximal cross-sectional area (Figure 5F) in SMET_B_ KO mice and control mice.

Immunohistochemistry (Figure S2) showed that SMET_B_ KO did not differ from controls in the amount of macrophage (Mac-2; SMET_B_ KO 2.7±0.9% versus Control 2.6±0.7% lesion area), α-smooth muscle actin (SMET_B_ KO 14.8±4.1% versus Control 19.9±3.8% lesion area), or collagen (SMET_B_ KO 9.7±3.1% versus Control 14.9±3.2% lesion area) staining in the neointimal lesions.

### SMET_B_ KO and Vascular Reactivity

In WT C57Bl/6J mice, EC removal from aortic rings abolished acetylcholine-mediated relaxation and enhanced the contractile response to phenylephrine but not to ET-1. EC removal from femoral arteries also abolished acetylcholine-mediated relaxation but had no effect on phenylephrine or ET-1 (Figure S3; Table S2). SMET_B_ KO had no effect on contractile responses to phenylephrine or ET-1, or acetylcholine-mediated relaxation in femoral arteries (Figure S4; Table S3).

### Induction of ET_B_-Mediated Contraction in Isolated Mesenteric Arteries

**Figure 6.**
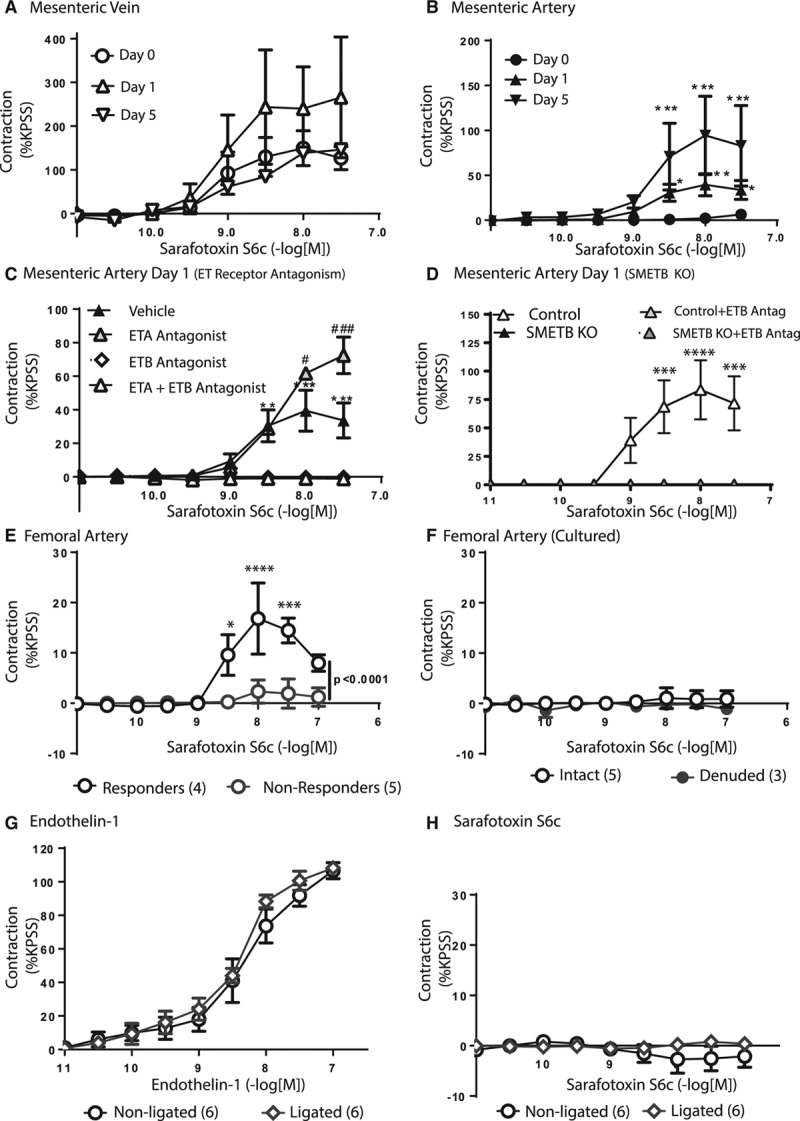
Impact of smooth muscle endothelin_B_ (SMET_B_ ) receptors on vascular function. **A**, Sarafotoxin S6c (S6c)-induced contraction in mesenteric veins (n=6) was not increased by incubation for 1 (n=3) or 5 (n=1) d in culture. **B**, Freshly isolated mesenteric arteries (n=6) did not respond to S6c, but contractions were induced by incubation in culture medium for 1 (n=7) or 5 (n=3) *P<0.05, **P<0.01, ***P<0.005 compared with d 0. **C**, S6c-mediated contraction of mesenteric arteries after 24 h in culture (n=7) was abolished by ET_B_ selective (A192621; 100 nmol/L; n=3) or mixed ET_A/B_ (BQ-123+A192621; n=3) antagonism, but not by ET_A_ receptor antagonism (BQ-123; 100 nmol/L; n=3); **P<0.01, ***P<0.005 compared with ET_B_ or ET_A/B_ antagonism; #*P*<0.05, ###*P*<0.005 compared with vehicle. **D**, In contrast to controls (n=4), S6c-mediated, A192621 (100 nmol/L)-sensitive contraction was not induced in mesenteric arteries from SMET_B_ knockout (KO) mice (n=4) by incubation in culture medium (24 h); ***P<0.005, ****P<0.001 compared with antagonists. **E**, Contractile responses to S6c were unreliable in femoral arteries—some failed to contract, whereas others produced small contractions. *P<0.05, ***P<0.005, ****P<0.001 compared with nonresponders. **F**, Incubation in culture did not induce S6c-mediated contraction in these arteries. Femoral arteries after ligation (28 d) contracted in response to endothelin-1 (**G**) but not to S6c (**H**). Data are mean±SEM (n=3 to 6). ET_A_ indicates endothelin_A_; and KPSS, potassium physiological salt solution.

ET-1–mediated contraction in mesenteric arteries from WT C57Bl/6J mice was shifted to the right by mixed ET_A/B_, or selective ET_A_, antagonism, but not by ET_B_ selective antagonism (Figure S5; Table S4). Unlike mesenteric veins (Figure 6A), mesenteric arteries freshly isolated from WT C57Bl/6J mice did not contract in response to S6c (Figure 6B).

Incubation in culture medium (≤5 days) can induce ET_B_-mediated contraction in rat arteries.^29^ Incubation of C57Bl/6J mesenteric veins in culture medium had no effect on S6c-mediated contraction (Figure 6A). In mesenteric arteries, incubation in culture medium selectively increased the contractile response to ET-1 (Table S5). Strikingly, S6c-mediated contraction was induced in isolated mesenteric arteries after incubation in culture medium (Figure 6B; Table S5), a response abolished by selective ET_B_, or mixed ET_A/B_, antagonism, but not by selective ET_A_ antagonism (Figure 6C; Table S6). Incubation of mesenteric arteries from SMET_B_ KO mice in culture medium did not induce S6c-mediated contraction (Figure 6D).

### No Induction of ET_B_-Mediated Contraction in Femoral Arteries

S6c-mediated contraction was variable in femoral arteries from WT C57Bl/6J mice: some contracted but others did not (Figure 6E). Neither incubation of femoral arteries in culture medium (24 hours; Figure 6F) nor lesion formation induced S6c-mediated contraction; femoral arteries isolated 28 days after ligation contracted in response to ET-1 (Figure 6G) but not to S6c (Figure 6H). Responses to acetylcholine, sodium nitroprusside, and phenylephrine were unaltered by lesion formation (Figure S6).

## Discussion

Tissue-specific knockout mice were generated to address the hypothesis that selective deletion of ET_B_ receptors from VSMC would impair arterial contraction, lower BP, and reduce neointimal lesion size. SMET_B_ KO attenuated S6c-mediated vascular and tracheal contraction, without altering other functional responses, but produced a modest (≈4 mm Hg) increase in BP. ET_B_-mediated contraction was not induced in femoral arteries after ligation, although injury-induced intimal lesion formation was unaffected by SMET_B_ KO. Key findings are summarized (Figure S7) and compared with the ECET_B_ KO (Table S7).

SMET_B_ KO was based on our generation of ECET_B_ KO,^22^ crossing mice expressing Cre-recombinase controlled by the SM-specific SM22 promoter^25^ with those bearing a floxed ET_B_ gene.^22^ This strategy was used to produce mice with SM-selective ET_A_ deletion,^4^ and renal collecting duct–selective ET_B_ deletion.^21^ It has also been used within our group to produce mice with SM-selective deletion of glucocorticoid receptor^31^ or 11β-hydroxysteroid dehydrogenase 1^32^ (with LacZ staining in Rosa26 reporter mice showing SM22cre expression in the blood vessels and heart but not in the brain, kidney, or adrenal gland). As with ECET_B_ KO,^22^ SMET_B_ KO mice were healthy. This contrasts with global ET_B_ deletion, which causes coat spotting and death from megacolon,^33^ requiring transgenic ET_B_ rescue in the enteric nervous system.^34^ Autoradiographic detection of ET_B_ receptors in lungs of SMET_B_ KO mice indicates maintained expression in EC (which was lost in ECET_B_ KO).^14^ This was supported by colocalization of immunoreactivity for ET_B_ with an EC marker (von Willebrand factor) in coronary arteries; absence of medial ET_B_ staining was consistent with deletion from SMCs. Polymerase chain reaction confirmed that ET_B_ had been deleted from aortic smooth muscle but not from heart, colon, or skeletal muscle (although direct evidence of ET_B_ deletion from tracheal, mesenteric vein, mesenteric, or femoral artery smooth muscle was not obtained using this technique). Functional investigations confirmed that SMET_B_-dependent responses were lost in the knockout, with the abolition of S6c-mediated contraction in mesenteric veins. Furthermore, induction of S6c-mediated contraction in mesenteric arteries incubated in culture medium (as in rat arteries^35^), was abolished by SMET_B_ KO (although these functional changes do not necessarily confirm selective SMET_B_ deletion). The failure to abolish S6c-induced contraction in trachea was unexpected and suggests either incomplete penetrance of SM22cre-mediated recombination or a role for ET_B_ receptors in other cells (eg, epithelium) in mediating tracheal contraction. Detection of the delta band in some ear clip samples may suggest deletion of the floxed gene in germ cells, which is a possible limitation with these mice. However, our F^+^/Cre0×F^+^/Cre0 crosses did not produce piebald mice (which inevitably would occur if germ-line recombination takes place). Therefore, the delta band during genotyping can only be explained by the presence of SMC in the ear clip preparations.

Selective deletion of ET_B_ from EC increased plasma ET-1^22^ because of impaired clearance.^14^ In contrast, SMET_B_ KO did not alter circulating ET-1, consistent with the proposal that ECET_B_ predominantly mediate ET-1 clearance.

Transgenic and pharmacological approaches suggest ET_B_ receptors regulate BP. Selective ET_B_ receptor antagonism,^20^ global ET_B_ deletion,^10^ and selective ET_B_ deletion from the collecting duct^21^ all increased (≈10–13 mm Hg) BP. Furthermore, ET_B_ receptors in peripheral ganglia can influence BP,^36^ suggesting that sympathetic activation accounts for ET_B_-induced hypertension.^37^ In contrast, BP was not elevated by ECET_B_ KO.^22^ The small (≈4 mm Hg) increase in BP, which persisted in SMET_B_ KO mice despite reduced heart rate, suggests that loss of SMET_B_ contributes to the increased BP induced by systemic ET_B_ antagonism^20^ or global ET_B_ deletion.^10^ However, it requires rejection of our hypothesis that ET_B_-mediated vascular contraction contributes to BP elevation. Indeed, our data support a role for extravascular ET_B_ (eg, in the kidney or peripheral ganglia) in regulating BP. This is supported by the demonstration that, as in ECET_B_ KO,^22^ salt-induced and ET_B_ antagonist–induced elevations of BP are unaltered by SMET_B_ KO. The mechanism underlying increased BP after SMET_B_ KO is not apparent but is unlikely to be a consequence of cre overexpression in SM because this did not alter baseline BP in SMET_A_ KO mice.^4^ Several possible explanations can be proposed. First, ET_B_ in VSMC may contribute to the clearance of ET-1 from tissue where it is preferentially secreted by EC, and where it acts. Therefore, SMET_B_ KO may cause ET-1 accumulation in the vascular wall, thus increasing ET-1–mediated vasoconstriction. Second, loss of SMET_B_ may upregulate ET_A_-mediated contraction. Third, SMET_B_ in the kidney may influence sodium homeostasis. Because SM22 may be expressed in perivascular fat precursors,^36^ loss of ET_B_ from perivascular fat may have caused developmental changes in vascular function that also contribute to elevated BP, but this has not been established. It is also not clear why basal diastolic blood pressure is selectively increased in the SMET_B_ KO, but this would be worthy of future investigation.

Increased BP in SMET_B_ KO mice could not be attributed to vascular dysfunction as, with the exception of responses to S6c, we found no evidence of impaired arterial relaxation or contraction. Weak ET_B_–mediated contraction in arteries is consistent with studies in rats.^35^ Preliminary investigations (unpublished data) indicated that S6c-induced contraction of freshly isolated murine arteries (femoral, mesenteric, and carotid) was not increased by NO synthase inhibition or by removal of the endothelium. These results indicate that we are not missing an ET_B_-mediated contraction that has been obscured by ET_B_-mediated relaxation. Induction of ET_B_-mediated contraction after incubation has been attributed to transcriptional regulation and MEK-ERK1/2 signaling.^22,38^ Abolition of this response in mesenteric arteries from SMET_B_ KO mice indicated that they lack both functional arterial ET_B_ receptors and the means to generate new receptors in this tissue.

ET_B_ upregulation in SMC, mediating vasoconstriction and proliferation in cardiovascular disease,^18,19^ might explain studies reporting similar benefit from mixed ET_A/B_ and selective ET_A_ antagonism in reducing lesion formation^23,39,40^ (despite the protective roles of ET_B_ in several tissues, eg, EC and kidney). However, the effectiveness of mixed ET_A/B_ and selective ET_A_ antagonism is likely to depend on the balance of ET_B_ receptor activity in EC and VSMC of an affected artery. Transient upregulation of ET_A_ and ET_B_ receptors has been demonstrated in arterial lesions.^41^ If these ET_B_ receptors contribute to lesion formation, then ET_B_ antagonism would be desirable. There was, however, no evidence of induced ET_B_-mediated contraction in mouse femoral arteries after ligation. Similar investigations could not be performed after wire injury because these vessels fail to contract ex vivo. It remains possible that ET_B_ upregulation occurs in other (eg, carotid) arteries.

Neointimal lesion formation is increased in rescued global ET_B_ knockout mice^10^ and in (spotted lethal) rats with global deletion of ET_B_,^24^ consistent an antiproliferative role for ET_B_ receptors. This is supported by demonstrations that ET_B_ receptor antagonism increases lesion size,^9,24^ with the suggestion that this is because of impaired ET_B_–mediated release of NO from EC. Indeed, increased lesion formation in mice with global ET_B_ deletion was partly attributed to impaired EC–derived NO release.^9^ In contrast, selective ECET_B_ deletion inhibited ET_B_-mediated relaxation^22^ but had no effect on arterial lesion formation.^9^ These results suggest, therefore, that the protective role of ET_B_ receptors is played by non-ECET_B_ receptors. The demonstration here that deletion of ET_B_ from the SMC does not alter lesion size indicates that, as with the receptors in EC,^9^ ET_B_ in SMC do not influence neointimal remodeling. This implicates nonvascular ET_B_ receptors, for example, in monocyte-derived macrophages, in the regulation of neointimal proliferation and atherosclerosis.^42^

In conclusion, we have demonstrated that selective ET_B_ receptors in SMC may contribute modestly to regulation of BP but have little influence on vascular contraction or neointimal proliferation. These data suggest that any detrimental role of SMET_B_ is minor (at least during normal physiology), and, therefore, that selective ET_A_ receptor antagonists (which preserve protective EC/renal ET_B_ signaling) should be preferred to mixed ET_A/B_ antagonists for treatment of vascular disease.

## Perspectives

Generation of mice with selective deletion of ET_B_ from SMC indicates that these receptors contribute to the increased BP induced by ET_B_ receptor antagonism but do not regulate arterial function or the fibroproliferative response to acute arterial injury. It would be interesting to determine whether ET_B_ in SMCs influence other cardiovascular diseases (eg, diabetic complications). Whether the data generated in these animals are replicated in mice with cardiovascular disease (eg, atherosclerosis), or in man, remains to be established. However, these results support the proposal that selective ET_A_ receptor antagonists may have advantages over mixed ET_A/B_ antagonists for combatting elevated BP or restenosis after revascularization.

## Acknowledgments

A192621 was a gift from AbbVie, United States.

## Sources of Funding

This work was funded by the British Heart Foundation (Project Grant PG/08/068/25461, P.W.F. Hadoke and D.J. Webb; Intermediate Clinical Research Fellowship FS/13/30/29994, N. Dhaun; and Centre of Research Excellence Award) and the Wellcome Trust (107715/Z/15/Z, A.P. Davenport and R.E. Kuc).

## Disclosures

D.J. Webb has provided advice to Abbott, AbbVie, AstraZeneca, Encysive, Pfizer, Retrophin, and Roche in relation to clinical development of endothelin receptor antagonists. K.M. Duthie received a Pfizer Young Investigator Award. N. Dhaun has received research grants from Pfizer.

## Supplementary Material

## Novelty and Significance

### What Is New?

- This study describes newly generated mice with selective endothelin_B_ (ET_B_) receptor deletion from smooth muscle. This was used to clarify the influence of smooth muscle ET_B_ receptors on (1) blood pressure, (2) arterial and venous contraction, and (3) arterial remodeling following injury.

### What Is Relevant?

- Generation of the knockout was necessary because ET_B_ receptors in vascular endothelial and smooth muscle cells cannot be distinguished pharmacologically. This work shows that ET_B_ receptors in smooth muscle have little influence on arterial function or neointimal remodeling but have a small suppressive effect on diastolic blood pressure. This is consistent with the proposal that selective endothelin_A_ (ET_A_) antagonism would be preferable to mixed ET_A_/ET_B_ antagonism for inhibiting arterial remodeling.

### Summary

Selective smooth muscle ET_B_ deletion indicated that these receptors play a minor role in regulation of BP but do not affect vascular function or remodeling. This suggests that, beyond endothelial cell ET_B_, ET_B_-dependent regulation of these processes is mediated by receptors in extravascular cells (eg, renal collecting ducts).

## References

[1] Kirkby NS, Hadoke PW, Bagnall AJ, Webb DJ (2008). The endothelin system as a therapeutic target in cardiovascular disease: great expectations or bleak house?. Br J Pharmacol.

[2] Davenport AP, Hyndman KA, Dhaun N, Southan C, Kohan DE, Pollock JS, Pollock DM, Webb DJ, Maguire JJ (2016). Endothelin.. Pharmacol Rev.

[3] Yanagisawa M, Kurihara H, Kimura S, Tomobe Y, Kobayashi M, Mitsui Y, Yazaki Y, Goto K, Masaki T (1988). A novel potent vasoconstrictor peptide produced by vascular endothelial cells.. Nature.

[4] Donato AJ, Lesniewski LA, Stuart D, Walker AE, Henson G, Sorensen L, Li D, Kohan DE (2014). Smooth muscle specific disruption of the endothelin-A receptor in mice reduces arterial pressure, and vascular reactivity and affects vascular development.. Life Sci.

[5] Komuro I, Kurihara H, Sugiyama T, Yoshizumi M, Takaku F, Yazaki Y (1988). Endothelin stimulates c-fos and c-myc expression and proliferation of vascular smooth muscle cells.. FEBS Lett.

[6] Li L, Chu Y, Fink GD, Engelhardt JF, Heistad DD, Chen AF (2003). Endothelin-1 stimulates arterial VCAM-1 expression via NADPH oxidase-derived superoxide in mineralocorticoid hypertension.. Hypertension.

[7] Amiri F, Virdis A, Neves MF, Iglarz M, Seidah NG, Touyz RM, Reudelhuber TL, Schiffrin EL (2004). Endothelium-restricted overexpression of human endothelin-1 causes vascular remodeling and endothelial dysfunction.. Circulation.

[8] Helset E, Sildnes T, Seljelid R, Konopski ZS (1993). Endothelin-1 stimulates human monocytes *in vitro* to release TNF-alpha, IL-1beta and IL-6.. Mediators Inflamm.

[9] Kirkby NS, Duthie KM, Miller E, Kotelevtsev YV, Bagnall AJ, Webb DJ, Hadoke PW (2012). Non-endothelial cell endothelin-B receptors limit neointima formation following vascular injury.. Cardiovasc Res.

[10] Murakoshi N, Miyauchi T, Kakinuma Y, Ohuchi T, Goto K, Yanagisawa M, Yamaguchi I (2002). Vascular endothelin-B receptor system *in vivo* plays a favorable inhibitory role in vascular remodeling after injury revealed by endothelin-B receptor-knockout mice.. Circulation.

[11] de Nucci G, Thomas R, D’Orleans-Juste P, Antunes E, Walder C, Warner TD, Vane JR (1988). Pressor effects of circulating endothelin are limited by its removal in the pulmonary circulation and by the release of prostacyclin and endothelium-derived relaxing factor.. Proc Natl Acad Sci USA.

[12] Hirata Y, Emori T, Eguchi S, Kanno K, Imai T, Ohta K, Marumo F (1993). Endothelin receptor subtype B mediates synthesis of nitric oxide by cultured bovine endothelial cells.. J Clin Invest.

[13] Fukuroda T, Fujikawa T, Ozaki S, Ishikawa K, Yano M, Nishikibe M (1994). Clearance of circulating endothelin-1 by ETB receptors in rats.. Biochem Biophys Res Commun.

[14] Kelland NF, Kuc RE, McLean DL, Azfer A, Bagnall AJ, Gray GA, Gulliver-Sloan FH, Maguire JJ, Davenport AP, Kotelevtsev YV, Webb DJ (2010). Endothelial cell-specific ETB receptor knockout: autoradiographic and histological characterisation and crucial role in the clearance of endothelin-1.. Can J Physiol Pharmacol.

[15] Goligorsky MS, Budzikowski AS, Tsukahara H, Noiri E (1999). Co-operation between endothelin and nitric oxide in promoting endothelial cell migration and angiogenesis.. Clin Exp Pharmacol Physiol.

[16] McCulloch KM, Docherty CC, Morecroft I, MacLean MR (1996). EndothelinB receptor-mediated contraction in human pulmonary resistance arteries.. Br J Pharmacol.

[17] Mickley EJ, Gray GA, Webb DJ (1997). Activation of endothelin ETA receptors masks the constrictor role of endothelin ETB receptors in rat isolated small mesenteric arteries.. Br J Pharmacol.

[18] Janakidevi K, Fisher MA, Del Vecchio PJ, Tiruppathi C, Figge J, Malik AB (1992). Endothelin-1 stimulates DNA synthesis and proliferation of pulmonary artery smooth muscle cells.. Am J Physiol.

[19] Dimitrijevic I, Edvinsson ML, Chen Q, Malmsjö M, Kimblad PO, Edvinsson L (2009). Increased expression of vascular endothelin type B and angiotensin type 1 receptors in patients with ischemic heart disease.. BMC Cardiovasc Disord.

[20] Fryer RM, Rakestraw PA, Banfor PN, Cox BF, Opgenorth TJ, Reinhart GA (2006). Blood pressure regulation by ETA and ETB receptors in conscious, telemetry-instrumented mice and role of ETA in hypertension produced by selective ETB blockade.. Am J Physiol Heart Circ Physiol.

[21] Ge Y, Bagnall A, Stricklett PK, Strait K, Webb DJ, Kotelevtsev Y, Kohan DE (2006). Collecting duct-specific knockout of the endothelin B receptor causes hypertension and sodium retention.. Am J Physiol Renal Physiol.

[22] Bagnall AJ, Kelland NF, Gulliver-Sloan F, Davenport AP, Gray GA, Yanagisawa M, Webb DJ, Kotelevtsev YV (2006). Deletion of endothelial cell endothelin B receptors does not affect blood pressure or sensitivity to salt.. Hypertension.

[23] Douglas SA, Louden C, Vickery-Clark LM, Storer BL, Hart T, Feuerstein GZ, Elliott JD, Ohlstein EH (1994). A role for endogenous endothelin-1 in neointimal formation after rat carotid artery balloon angioplasty. Protective effects of the novel nonpeptide endothelin receptor antagonist SB 209670.. Circ Res.

[24] Kitada K, Yui N, Matsumoto C, Mori T, Ohkita M, Matsumura Y (2009). Inhibition of endothelin ETB receptor system aggravates neointimal hyperplasia after balloon injury of rat carotid artery.. J Pharmacol Exp Ther.

[25] Holtwick R, Gotthardt M, Skryabin B, Steinmetz M, Potthast R, Zetsche B, Hammer RE, Herz J, Kuhn M (2002). Smooth muscle-selective deletion of guanylyl cyclase-A prevents the acute but not chronic effects of ANP on blood pressure.. Proc Natl Acad Sci USA.

[26] Davenport AP, Kuc RE (2002). Radioligand binding assays and quantitative autoradiography of endothelin receptors.. Methods Mol Biol.

[27] Ling L, Kuc RE, Maguire JJ, Davie NJ, Webb DJ, Gibbs P, Alexander GJ, Davenport AP (2012). Comparison of endothelin receptors in normal versus cirrhotic human liver and in the liver from endothelial cell-specific ETB knockout mice.. Life Sci.

[28] Opgenorth TJ, Adler AL, Calzadilla SV, Chiou WJ, Dayton BD, Dixon DB, Gehrke LJ, Hernandez L, Magnuson SR, Marsh KC, Novosad EI, Von Geldern TW, Wessale JL, Winn M, Wu-Wong JR (1996). Pharmacological characterization of A-127722: an orally active and highly potent ETA-selective receptor antagonist.. J Pharmacol Exp Ther.

[29] Adner M, Uddman E, Cardell LO, Edvinsson L (1998). Regional variation in appearance of vascular contractile endothelin-B receptors following organ culture.. Cardiovasc Res.

[30] Kirkby NS, Low L, Seckl JR, Walker BR, Webb DJ, Hadoke PW (2011). Quantitative 3-dimensional imaging of murine neointimal and atherosclerotic lesions by optical projection tomography.. PLoS One.

[31] Rog-Zielinska EA, Thomson A, Kenyon CJ, Brownstein DG, Moran CM, Szumska D, Michailidou Z, Richardson J, Owen E, Watt A, Morrison H, Forrester LM, Bhattacharya S, Holmes MC, Chapman KE (2013). Glucocorticoid receptor is required for foetal heart maturation.. Hum Mol Genet.

[32] White CI, Jansen MA, McGregor K, Mylonas KJ, Richardson RV, Thomson A, Moran CM, Seckl JR, Walker BR, Chapman KE, Gray GA (2016). Cardiomyocyte and vascular smooth muscle-independent 11β-hydroxysteroid dehydrogenase 1 amplifies infarct expansion, hypertrophy, and the development of heart failure after myocardial infarction in male mice.. Endocrinology.

[33] Hosoda K, Hammer RE, Richardson JA, Baynash AG, Cheung JC, Giaid A, Yanagisawa M (1994). Targeted and natural (piebald-lethal) mutations of endothelin-B receptor gene produce megacolon associated with spotted coat color in mice.. Cell.

[34] Gariepy CE, Williams SC, Richardson JA, Hammer RE, Yanagisawa M (1998). Transgenic expression of the endothelin-B receptor prevents congenital intestinal aganglionosis in a rat model of Hirschsprung disease.. J Clin Invest.

[35] Adner M, Geary GG, Edvinsson L (1998). Appearance of contractile endothelin-B receptors in rat mesenteric arterial segments following organ culture.. Acta Physiol Scand.

[36] Chang L, Villacorta L, Li R, Hamblin M, Xu W, Dou C, Zhang J, Wu J, Zeng R, Chen YE (2012). Loss of perivascular adipose tissue on peroxisome proliferator-activated receptor-γ deletion in smooth muscle cells impairs intravascular thermoregulation and enhances atherosclerosis.. Circulation.

[37] Fink G, Li M, Lau Y, Osborn J, Watts S (2007). Chronic activation of endothelin B receptors: new model of experimental hypertension.. Hypertension.

[38] Zhang W, Li XJ, Zeng X, Shen DY, Liu CQ, Zhang HJ, Xu CB, Li XY (2012). Activation of nuclear factor-κB pathway is responsible for tumor necrosis factor-α-induced up-regulation of endothelin B2 receptor expression in vascular smooth muscle cells *in vitro*.. Toxicol Lett.

[39] Azuma H, Sato J, Masuda H, Goto M, Tamaoki S, Sugimoto A, Hamasaki H, Yamashita H (1999). ATZ1993, an orally active and novel nonpeptide antagonist for endothelin receptors and inhibition of intimal hyperplasia after balloon denudation of the rabbit carotid artery.. Jpn J Pharmacol.

[40] Sanmartín M, Fernández-Ortiz A, Fantidis P, Aragoncillo P, Fernández-Durango R, Rollín R, Alfonso F, Hernández R, Escaned J, Macaya C (2003). Effects of bosentan on neointimal response following coronary angioplasty.. Eur J Clin Invest.

[41] Wang X, Douglas SA, Louden C, Vickery-Clark LM, Feuerstein GZ, Ohlstein EH (1996). Expression of endothelin-1, endothelin-3, endothelin-converting enzyme-1, and endothelin-A and endothelin-B receptor mRNA after angioplasty-induced neointimal formation in the rat.. Circ Res.

[42] Haug C, Schmid-Kotsas A, Zorn U, Schuett S, Gross HJ, Gruenert A, Bachem MG (2001). Endothelin-1 synthesis and endothelin B receptor expression in human coronary artery smooth muscle cells and monocyte-derived macrophages is up-regulated by low density lipoproteins.. J Mol Cell Cardiol.

