# Supplementary material for: Smooth Muscle Endothelin B Receptors Regulate Blood Pressure but Not Vascular Function or Neointimal Remodeling
Source: Hypertension. 2017 Jan 11;69(2):275–85. doi: 10.1161/HYPERTENSIONAHA.115.07031 (PMC5222555; doi:10.1161/HYPERTENSIONAHA.115.07031)
Supplement: Supplementary file 1 [file hyp-69-275-s001.docx]

Smooth muscle endothelin B receptors regulate blood pressure but not vascular function or neointimal remodelling

**Supplementary Information**

Eileen Miller^1^, Alicja Czopek^1^, Karolina M Duthie^1^, Nicholas S Kirkby^1^, Elisabeth E Fransen van de Putte^1^, Sibylle Christen^2^, Robert A. Kimmitt^1^, Rebecca Moorhouse, Raphael FP Castellan^1^, Yuri V Kotelevtsev^3^, Rhoda E Kuc^4^, Anthony P Davenport^4^, Neeraj Dhaun^1^, David J Webb^1^ and Patrick WF Hadoke^1^

*^1^Centre for Cardiovascular Science, University of Edinburgh, Edinburgh, UK, ^2^University of Basel, Switzerland, Stem Cell Genome Modification Laboratory, ^3^Skolkovo Institute of Science and Technology* *Novaya St. 100, 143025, Skolkovo , Russian Federation, ^4^Division of Experimental Medicine & Immunotherapeutics (EMIT), Addenbrooke's Hospital, Hills Road, Cambridge CB2 20QQ.*

**Short Title:** ET_B_ deletion, BP and arterial remodelling

***Animals***

Mice with VSMC selective deletion of ET_B_ receptors were generated based on a strategy described previously to produce EC ETB KO mice^1^. Homozygous (Flox/Flox) ET_B_ mice (background: 50% 129/Ola and 50% BKW) were crossed with SM22-Cre transgenic mice and backcrossed to C57Bl/6J for 4-6 generations. As for previous studies^1,2^ floxed, Cre-negative littermates (ET_B_^f/f^) were used as experimental controls for SM-specific ET_B_ deficient mice (SM ET_B_ KO). Genotyping to identify wild type and recombined alleles was performed by PCR^1^ and the SM22-Cre transgene was detected as described^3^. All mice were given free access to tap water and standard mouse chow. Mice were housed according to United Kingdom Home Office recommendations at 22°C with 12-hour diurnal light/dark cycles. All procedures were performed under the provisions of the Animals Scientific Procedures Act (1986) and with the approval of the local ethics committee.

***Autoradiography***

After euthanasia, SM ET_B_ KO mice and controls (n=3/group) were rapidly frozen at -70^o^C, torsos were mounted in a cryostat and consecutive 30µm longitudinal sections were cut to encompass the major thoracic and abdominal organs. Sections were thaw-mounted onto gelatin-coated slides^4^ and ligand binding assays performed as described^5^. Briefly, consecutive sections were incubated with 0.25nM of the ET_A_ selective ligand [^125^I]PD151242 or 0.25nM of the ET_B_ selective ligand [^125^I]BQ2030 (Amersham Bioscience, GE Healthcare, UK). Non-specific binding was determined by co-incubating adjacent sections with the ligand and corresponding excess unlabelled peptide. Slides with calibrated standards were exposed to Kodak MR-1 autoradiography film for 4 days before being developed.

***Immunohistochemistry***

Immunohistochemical identification of ET_B_ receptors was performed as previously described^6^. Site directed antisera were raised in rabbits to the sequence ET_B_(302-313), as described^7^. Briefly, whole body tissue sections (15 μm) were dried overnight at room temperature and fixed in ice-cold acetone for 10 minutes. Three knock-out and three control mice were examined.

Slides were incubated with 5% non-immunised donkey serum (DS) in phosphate-buffered saline (PBS) for 1 hour at room temperature to block non-specific protein interactions and then incubated overnight at 4 °C with primary rabbit anti-ET_B_ (1:50) antiserum and primary goat anti-von Willebrand factor (1:50) PBS/0.1% Tween-20/3% DS. Slides were then washed (3×5 minutes) in cold 1% PBS/0.1% Tween-20 before incubation for 1 hour at room temperature with Alexa Fluor 488 conjugated donkey anti-rabbit (1:200), Alexa Fluor 568 conjugated donkey anti-goat (1:100) secondary antibodies and Hoechst (1:100) diluted in 1% PBS/0.1% Tween-20/3% DS. Tissue sections were washed again (3×5 minutes) in cold 1% PBS/0.1% Tween-20 and mounted with ProLong Gold (Invitrogen). Confocal imaging was performed using a Leica TCS-NT-UV confocal laser-scanning microscope (Leica Microsystems, Heidelberg,Germany).

**Ex vivo *analysis of ET_B_-mediated contraction***

Functional analyses were performed using isolated mouse trachea, aorta, femoral arteries, and 1^st^ order mesenteric arteries and veins, as described^8^. The endothelium was removed from some aortic rings by rubbing the luminal surface with a wire. Some arteries and veins were incubated in serum-free medium (DMEM) for 1-5 days before functional analysis to induce ET_B_-mediated contraction, after the method of Adner *et al.*^9^. Briefly, rings (~2mm in length) of trachea, femoral artery, or mesenteric artery or vein were suspended on two intraluminal 40 µm tungsten wires in a myograph (model 610M Multi-myograph; JP Trading, Aarhus, Denmark) chamber. These rings were equilibrated at their optimum resting force (trachea 2mN; aorta 7.36mN, femoral artery, 8mN; mesenteric artery 3mN; mesenteric vein, 1mN) in physiological salt solution (PSS; 119 mM NaCl, 14.9 mM NaHCO_3_, 4.7 mM KCl, 1.18 mM KH_2_PO_4_, 1.17 mM MgSO_4_, 1.6 mM CaCl_2_, 0.026 mM EDTA, 5.5 mM glucose), aerated (95% O_2_, 5% CO_2_) and maintained at 37°C. Each ring was then exposed to a high potassium (125mM) PSS (KPSS. Cumulative concentration-response curves were obtained to phenylephrine (PE, 1x10^-9^-3x10^-3^M), ET-1 (1x10^-11^-3x10^-5^M), acetylcholine (1x10^-9^-3x10^-3^M) or sarafotoxin 6c (S6c; 1x10^-11^-3x10^-5^M), as required. Some rings were incubated with an ET_A_ antagonist (BQ123; 100nM), an ET_B_ antagonist (A192621; 100nM) or a mixture of the two antagonists 20 min before acquiring cumulative concentration-response curves. All responses were measured and recorded with Powerlab software.

***Measurement of BP***

Male SM ET_B_ KO mice and age-matched controls (n=8/ group) were caged singly and maintained on standard mouse chow (7 days) before measurement of BP and heart rate using telemetry, as described previously^1^. Briefly, under isoflurane anesthesia, a telemetry catheter was inserted into the left carotid artery and the transmitter device (Data Sciences) secured in the left flank. Mice were allowed to recover and were maintained on standard chow (7 days), high (7.6%) salt diet (7 days), then high salt plus ET_B_ antagonist (SB192621; 30/mg/kg/day in drinking water, 7 days). Systolic and diastolic BP and heart rate were recorded in unrestrained mice (for 5 min every 30 min) as described previously^1^ and analyzed using the Powerlab data acquisition system. Average blood pressures over each 5 min period (48 measurements/ day) were used to calculate the 24 h average BP.

***Femoral artery injury***

Intra-luminal injury was performed as described^2^. Briefly, under inhaled isoflurane anaesthesia (induction 5%, maintenance 2-3%) a 0.014” diameter straight-sprung angioplasty guide wire was advanced ~1.5cm proximally into the isolated femoral artery through an arteriotomy in the popliteal branch. After withdrawal, the popliteal branch was ligated to allow re-perfusion of the injured femoral artery. Non-denuding injury was achieved by ligation of the right femoral artery at the femero-popliteal bifurcation^2^. Peri-operative analgesia was provided by administration of buprenorphine (0.05mg/kg buprenorphine s.c.; Alstoe Animal Health, UK). Mice were then allowed to recover (28 days) to allow lesion development.

***Perfusion fixation***

After the recovery period mice were killed by perfusion fixation. Under terminal anaesthesia (sodium pentobarbital, Ceva Animal Health, UK, 60 mg/kg; i.p.), thoracotomy and transverse sternotomy were performed to allow introduction of a 23-gauge needle into the left ventricle. Phosphate buffered saline containing heparin (Leo Laboratories, UK, 10 U/ml) was administered (6 ml/min) via the left ventricle and an incision was made in the right ventricle to allow perfusate to wash through. Once blood was washed out, 10% neutral buffered formalin (Sigma, UK) was perfused until adequate fixation occurred (indicated by the development of rigidity of the body). Following perfusion fixation, femoral arteries, liver, heart and kidneys were removed. Organs were weighed and all tissues were left in formalin for a further 48h before processing to paraffin for histological assessment.

***Optical projection tomography***

Non-destructive 3-dimensional assessment of lesions was performed using optical projection tomography (OPT), as described^10,11^. Briefly, vessels were embedded in agarose and optically cleared in benzyl alcohol/ benzyl benzoate. Intrinsic fluorescent emission was imaged (excitation filter: 425/40 nm; emission filter: 475 nm low pass) using a Bioptonics 3001 tomograph. Data were reconstructed by filtered back projection using NRecon software (Skyscan, Belgium) and volumetric measurements generated by semi-automated tracing of the internal elastic lamina and the neointima distinguished from the lumen using a grey level threshold.

***Histological assessment of neointimal lesions***

Sections (4 µm) were cut from paraffin-embedded femoral arteries at 80 µm intervals with a Leitz 1512 microtome (Leica microsystems, Germany), and mounted onto Superfrost glass slides. Every tenth slide was selected for staining (Shandon Varistain Gemini automated slide stainer) with United States Trichrome, as described^12^. Images were taken using an Axioskop KS300 stage microscope (Carl Zeiss Inc., UK) and a CCD camera (photometrics USA) with a liquid crystal filter (MicroColour, LRI, Inc, USA). Image analysis was performed using MCID basic 7.0 software (Imaging Research, USA). The location of the maximal lesion was determined and serial sections used for compositional analysis, including picro-sirius staining for the quantification of collagen content.

***Immunohistochemistry***

De-waxed and re-hydrated sections were blocked with goat serum before incubation with primary antibodies to α-smooth muscle actin (1:400; 30 min; Sigma, UK) and Mac2 (1:6000; overnight; Cedarlane, USA). Sections were then washed and incubated with a secondary antibody (goat anti-mouse or goat anti-rat, respectively; 1:400, 30 min; Vector Labs, UK). This was followed by incubation with streptavidin-conjugated horseradish peroxidase Extravidin; 30 min; Sigma, UK). Slides were developed by addition of 3,3-diaminobenzidine (DAB peroxidase staining kit, Vector Lab, UK) for 1 min. Images were taken as before and analysed with Image J software.

References

1. Bagnall AJ, Kelland NF, Gulliver-Sloan F, Davenport AP, Gray GA, Yanagisawa M, Webb DJ, Kotelevtsev YV. Deletion of endothelial cell endothelin B receptors does not affect blood pressure or sensitivity to salt. *Hypertension*. 2006;48:286-293.

2. Kirkby NS, Duthie KM, Miller E, Kotelevtsev YV, Bagnall AJ, Webb DJ, Hadoke PWF. Non-endothelial cell endothelin-B receptors limit neointima formation following vascular injury. *Cardiovasc Res.* 2012;95:19-28.

3. Holtwick R, Gotthardt M, Skryabin B, Steinmetz M, Potthast R, Zetsche B, Hammer RE, Herz J, Kuhn M. Smooth muscle-selective deletion of guanylyl cyclase-A prevents the acute but not chronic effects of ANP on blood pressure. *Proc Natl Acad Sci USA.* 2002;99:7142–147.

4. Davenport AP, Kuc RJ. Radioligand binding assays and quantitative autoradiography of endothelin receptors. *Methods Mol Cell Biol.* 2002;206:45-70.

5. Kelland NF, Kuc RE, McLean DL, Azfer A, Bagnall AJ, Gray GA, Gulliver-Sloan FH, Maguire JJ, Davenport AP, Kotelevtsev YV, Webb DJ*.* Endothelial cell specific ETB receptor knockout: autoradiographic and histological characterisation and crucial role in the clearance of endothelin-1. *Can J Physiol Pharmacol*. 2010;88:644-651.

6. Ling, L, Kuc, RE, Maguire, JJ, Davie, NJ, Webb, DJ, Gibbs, P, Alexander, GJM, Davenport, AP. Comparison of endothelin receptors in normal versus cirrhotic human liver and in the liver from endothelial cell-specific ET_B_ knockout mice. *Life Sciences.* 2012;91:716-722.

7. Kuc R, Davenport AP Comparison of endothelin-A and endothelin-B receptor distribution visualized by radioligand binding versus immunocytochemical localization using subtype selective antisera. *J Cardiovasc Pharmacol* 2004; 44 Suppl 1:S224-226.

8. Opgenorth TJ, Adler AL, Calzadilla SV, Chiou WJ, Dayton BD, Dixon DB, Gehrke LJ, Hernandez L, Magnusson SR, Marsh KC, Novosad, EI, von Geldern TW, Wessale GL, Winn M, Wu-Wong JR*.* Pharmacological characterisation of A-127722: an orally active and highly potent ET(A)-selective receptor antagonist. *J Pharmacol Exp Ther.* 1996;276:473-481.

9. Adner M, Uddmann E, Cardell LO, Edvinsson L. Regional variation in appearance of vascular contractile endothelin-B receptors following organ culture. *Cardiovascular Res.* 1998;37:254-262.

10. Kirkby, NS, Low, L, Riemersma, RA, Seckl, JR, Walker, BR, Webb, DJ, Hadoke, PWF. Quantitative 3-dimensional imaging of murine neointimal and atherosclerotic lesions. *PLoS ONE.* 2011;6 (2):*e16906*:1-8.*.*

11. Kirkby NS, Low, L, Wu, J, Miller, E, Seckl, JR, Walker, BR, Webb, DJ, Hadoke, PWF Generation and 3-dimensional quantitation of arterial lesions in mice using optical projection tomography. *J Vis Exp*. 2015; 99: 10.3791/50627.

12. Hadoke PWF, Wainwright CL, Wadsworth RM, Butler KD, Giddings M.J. Characterisation of the morphological and functional alterations in rabbit subclavian artery subjected to balloon angioplasty. *Coron Art Dis.* 1995; 6: 403-415.

**Supplementary Table S1.** Deletion of ET_B_ from smooth muscle did not alter body or organ weights.

| **Age & Weights** | **Wild Type** | **SMET_B_ KO** |
| --- | --- | --- |
| Age (weeks) | 20.0 ± 1.4 | 20.8 ± 1.6 |
| Body Weight (g) | 31.6 ± 0.8 | 32.1 ± 0.6 |
| Heart (% Body Weight) | 0.56 ± 0.03 | 0.56 ± 0.02 |
| Liver (% Body Weight) | 5.08 ± 0.15 | 4.88 ± 0.18 |
| Right Kidney (% Body Weight) | 0.72 ± 0.03 | 0.74 ± 0.03 |
| Right Kidney (% Body Weight) | 0.68 ± 0.02 | 0.71 ± 0.03 |
| Lung (% Body Weight) | 0.54 ± 0.03 | 0.56 ± 0.02 |

*Data are mean ± s.e. mean, n=7.*

**Supplementary Table S2.** Impact of endothelial cell removal on function of aorta and femoral arteries from control mice.

| **Drug** | **Measurement** | **Aorta**  **(Intact)** | **Aorta**  **(Denuded)** | **Femoral Artery**  **(Intact)** | **Femoral Artery (Denuded)** |
| --- | --- | --- | --- | --- | --- |
| PE | N | 3 | 3 | 3 | 3 |
|  | pD_2_ | 7.1 ± 0.2 | 7.3 ± 0.2 | 5.8 ± 0.1 | 6.5 ± 0.1* |
|  | Emax (%) | 80.3 ± 5.5 | 199.8 ± 11.4* | 100.3 ± 6.2 | 94.6 ± 4.5 |
|  | Emax (mN/mm) | 3.92 ± 0.34 | 3.48 ± 0.19 | 2.35 ± 0.20 | 1.61 ± 0.14 |
|  |  |  |  |  |  |
| ACh | N | *3* | *3* | *3* | *3* |
|  | -logIC_50_ | 7.4 ± 0.4 | 7.9 ± 1.0 | 7.4 ± 0.2 | 6.5 ± 6.1 |
|  | Emax (%) | 62.7 ± 7.1 | 31.5 ± 7.3 | 108.6 ± 5.7 | 19.5 ± 11.8* |
|  |  |  |  |  |  |
| ET-1 | N | 3 | 3 | 3 | 3 |
|  | pD_2_ | 8.5 ± 0.4 | 8.0 ± 0.2 | 8.2 ± 0.2 | 8.4 ± 0.1 |
|  | Emax (%) | 53.4 ± 8.4 | 74.3 ± 4.7 | 95.2 ± 4.5 | 89.6 ± 4.2 |
|  | Emax (mN/mm) | 1.78 ± 0.42 | 1.23 ± 0.20 | 2.51 ± 0.36 | 2.35 ± 0.46 |
|  |  |  |  |  |  |
| KPSS | N | 4 | 4 | 4 | 4 |
|  | Emax (mN/mm) | 2.91 ± 0.58 | 2.03 ± 0.43 | 3.21 ± 1.36 | 2.56 ± 1.23 |

Data are mean+s.e.mean. ACh, acetylcholine, ET-1, endothelin-1, KPSS, high (125mM) potssium physiological salt solution. PE, phenylephrine. *P<0.05 compared with intact artery. Emax, maximum contraction; pD_2_, -log EC_50_.

**Supplementary Table S3** Impact of deletion of ET_B_ from vascular smooth muscle on functional responses of femoral arteries.

| **Drug** | **Measurement** | **Wild Type (Intact)** | **Wild Type (Denuded)** | **SM ET_B_ KO (Intact)** | **SM ET_B_ KO (Denuded)** |
| --- | --- | --- | --- | --- | --- |
| **PE** | N | 6 | 7 | 7 | 7 |
|  | pD_2_ | 5.7 ± 0.1 | 6.0 ± 0.1 | 5.6 ± 0.1 | 6.1 ± 0.1 |
|  | Emax(%) | 88.9 ± 3.2 | 94.2 ± 3.2 | 91.9 ± 4.4 | 102.8 ± 3.8 |
|  | Emax (mN/mm) | 3.99 ± 0.35 | 2.05 ± 0.26 | 3.48 ± 0.25 | 2.37 ± 0.20 |
|  |  |  |  |  |  |
| **ACh** | N | 5 | 6 | 6 | 6 |
|  | -logIC_50_ | 8.0 ± 0.6 | 6.8 ± 0.4^†^ | 7.9 ± 0.5 | 7.2 ± 0.6* |
|  | Emax(%) | 120.1 ± 10.2 | 57.8 ± 6.4* | 120.9 ± 9.6 | 22.5 ± 3.2^‡^ |
|  |  |  |  |  |  |
| **ET-1** | N | 6 | 7 | 7 | 7 |
|  | pD_2_ | 7.8 ± 0.2 | 7.8 ± 0.3 | 7.9 ± 0.2 | 8.0 ± 0.2 |
|  | Emax(%) | 97.4 ± 7.9 | 112.9 ± 3.5 | 105.4 ± 5.8 | 130.3 ± 5.4 |
|  | Emax (mN/mm) | 4.03 ± 0.42 | 2.46 ± 0.40 | 4.04 ± 0.33 | 2.99 ± 0.27 |
|  |  |  |  |  |  |
| **KPSS** | N | 6 | 7 | 7 | 7 |
|  | Emax (mN/mm) | 4.44 ± 0.81 | 1.99 ± 0.64* | 3.84 ± 0.52 | 2.38 ± 0.54 |

Data are mean+s.e.mean. SM ETB KO, selective deletion of the endothelin B receptor from smooth muscle. ACh, acetylcholine, ET-1, endothelin-1, KPSS, high (125mM) potassium physiological salt solution. PE, phenylephrine. *P<0.05, ^†^P<0.01, ^‡^P<0.005 compared with intact artery. Emax, maximum contraction; pD_2_, -log EC_50_.

**Supplementary Table S4.** Impact of endothelin receptor antagonism on endothelin-1-mediated contraction of murine mesenteric arteries.

|  | **Antagonist** | | | |
| --- | --- | --- | --- | --- |
| **Measurement** | **Vehicle** | **ET_A_** | **ET_B_** | **ET_A/B_** |
| N | 7 | 3 | 3 | 3 |
| pD_2_ | 8.2+0.5 | 7.4+0.2* | 8.0+0.1 | 7.0+0.1* |
| Emax (mN/mm) | 2.4+0.7 | 2.4+1.3 | 3.4+0.5 | 2.3+1.0 |
| Emax (% KPSS) | 105+17 | 102+21 | 111+10 | 75+30 |

Data are mean+s.e.mean. *P<0.05 compared with Vehicle. Emax, maximum contraction; pD_2_, -log EC_50_.

**Supplementary Table S5.** Incubation induces ET_B_-mediated contraction in mouse mesenteric arteries.

| **Drug** | **Measurement** | **Incubation** | | |
| --- | --- | --- | --- | --- |
|  |  | **Day 0** | **Day 1** | **Day 5** |
| **KPSS** | N | 7 | 7 | 3 |
|  | Emax (mN/mm) | 2.47+0.80 | 2.42+0.68 | 1.40+1.03 |
| **PE** | N | 7 | 7 | 3 |
|  | pD_2_ | 5.90+0.25 | 5.71+0.24 | 5.65+0.44 |
|  | Emax (mN/mm) | 2.33+0.70 | 2.89+1.30 | 1.04+1.24 |
|  | Emax (% KPSS) | 97.8+15.6 | 114.1+25.1 | 111.7+91.7 |
| **S6c** | N | 7 | 7 | 3 |
|  | pD_2_ | --- | 8.75+0.19 | 8.73+0.05 |
|  | Emax (mN/mm) | 0.13+0.16 | 1.13+1.08* | 0.82+0.60^†^ |
|  | Emax (% KPSS) | 6.4+7.6 | 40.2+32.6* | 94.5+75.4^†^ |
| **ET-1** | N | 7 | 5 | 3 |
|  | pD_2_ | 8.24+0.53 | 8.50+0.40 | 8.54+0.25 |
|  | Emax (mN/mm) | 2.45+0.73 | 3.79+0.88* | 1.35+1.22* |
|  | Emax (% KPSS) | 105.4+17.8 | 164.1+16.4* | 132.8+87.6 |

Data are mean+s.e.mean.. ET-1, endothelin-1, KPSS, high (125mM) potassium physiological salt solution, PE, phenylephrine, S6c, sarafotoxin s6c. *P<0.05 compared with Day 0. ^†^P<0.05 compared with Day 1. Emax, maximum contraction; pD_2_, -log EC_50_.

**Supplementary Table S6.** Impact of endothelin receptor antagonism on sarafotoxin s6c-mediated contraction of murine mesenteric arteries.

|  |  | **Antagonist** | | | |
| --- | --- | --- | --- | --- | --- |
| **Timepoint** | **Measurement** | **Vehicle** | **ET_A_** | **ET_B_** | **ET_A/B_** |
| Day 0 | N | 7 | 3 | 3 | 3 |
|  | pD_2_ | NC | NC | NC | NC |
|  | Emax (mN/mm) | 0.13+0.16 | 0.01+0.01 | 0.03+0.03 | 0.04+0.04 |
|  | Emax (% KPSS) | 6.4+7.6 | 0.90+1.3 | 1.0+06 | 1.2+1.2 |
| Day 1 | N | 7 | 3 | 3 | 3 |
|  | pD_2_ | 8.75+0.19 | 8.41+0.17* | NC | NC |
|  | Emax (mN/mm) | 1.13+1.08 | 2.63+0.90 | 0.01+0.01 | 0.00+0.01 |
|  | Emax (% KPSS) | 40.2+32.6 | 73.3+17.75 | 0.7+0.4* | 0.4+0.4* |

Day 0, fresh arteries; Day 1, 24 h incubation. Data are mean+s.e.mean.. NC, not calculated. *P<0.05 compared with Vehicle. Emax, maximum contraction; pD_2_, -log EC_50_.

**Supplementary Table S7.** The impact of vascular cell selective deletion of murine vascular ET_B_ receptors (compared with wild type controls).

| **Measurement** | **EC ET_B_ KO*** | **SMC ET_B_ KO** |
| --- | --- | --- |
| Body Weight | **NSD^1, 2, 3, 4^** | **NSD** |
| Organ Weight | **NSD^1, 2,4^** | **NSD** |
| Plasma [ET-1] |  |  |
| Basal | **Increased^2^** | **NSD** |
| High salt diet + ET_B_ antagonist |  | **NSD** |
| Blood Pressure (basal) |  |  |
| Basal | **NSD^2^** | **Small (4mmHg) Increase** |
| High salt diet | **NSD^2^** | **Small (4mmHg) Increase** |
| High salt diet + ET_B_ antagonist. | **NSD^2^** | **NSD** |
| Heart Rate | **NSD^2^** | **Reduced** |
| Neointimal Proliferation | **NSD^1, 4^** | **NSD** |
| ET_B_-mediated contraction |  |  |
| Trachea | **NSD^1, 2,4^** | **Reduced** |
| Mesenteric Vein | **N/A** | **Abolished** |
| Mesenteric Artery | **N/A** | **Induction Abolished** |
| Femoral Artery | **N/A** | **N/A** |
| ACh-mediated relaxation |  |  |
| Aorta | **Impaired^2^** |  |
| Femoral Artery | **NSD^1, 4^** | **NSD** |
| ET-1-mediated contraction |  |  |
| Femoral Artery | **NSD^1, 4^** | **NSD** |
| PE-mediated contraction |  |  |
| Femoral Artery | **NSD^1,4^** | **NSD** |

From previous investigations. ^1^Kirkby *et al.*, 2012, ^2^Bagnall *et al.*, 2006; ^3^Kelland *et al.*, 2010; ^4^Kirkby, N.S. PhD Thesis Edinburgh 2009. ACh, acetylcholine; ET-1, endothelin-1; PE, phenylephrine; ET_B_, endothelin B receptor; NSD, No significant difference compared with Wild Type; N/A, not assessed.

**Supplementary Figure S1**


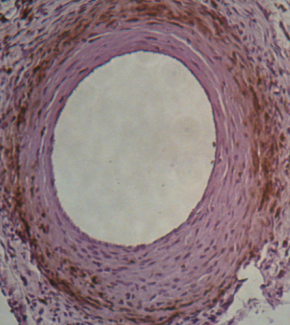

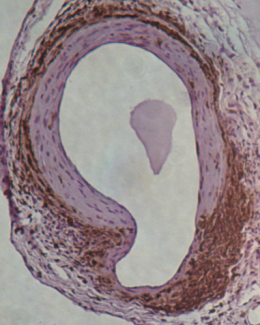


**Miller *et al.***

(a) Smooth Muscle Actin (αSMA)

(b) Macrophages (Mac 2)

(c) Collagen (picrosirius red)

SMET_B_ KO

Wild Type


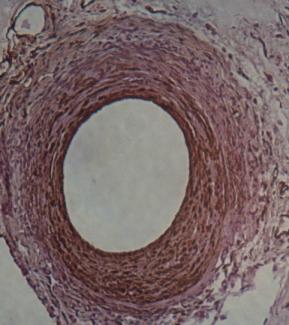

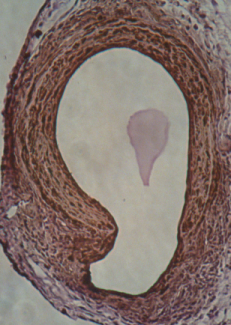


SMET_B_ KO

Wild Type

L

NI

L

NI

**Supplementary Figure S1. Identification of smooth muscle, macrophages and collagen in neointimal lesions.**

Compositional analysis of neointimal lesions induced by wire injury in smooth muscle selective ET_B_ KO (SMET_B_KO) and wild type mouse femoral arteries showing the presence of immunoreactivity for (a) Smooth muscle actin (brown) and (b) Macrophages (Mac 2; brown). (c) Picrosirius red staining (pink) identified collagen in the neointima and media. Scale bar = 100μm. L, Lumen, NI, neointima.


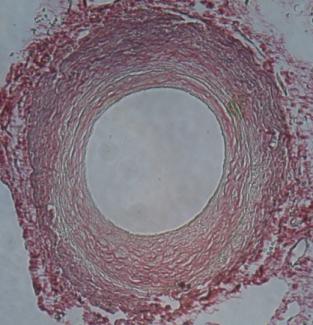

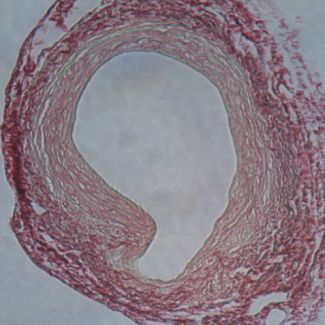


SMET_B_ KO

Wild Type


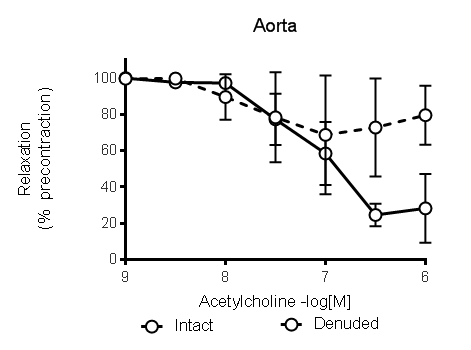

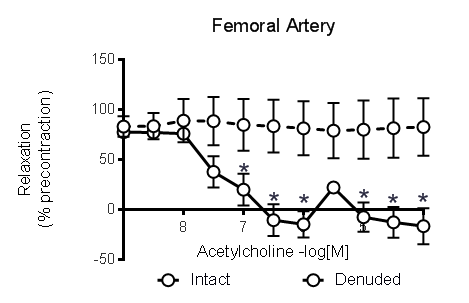

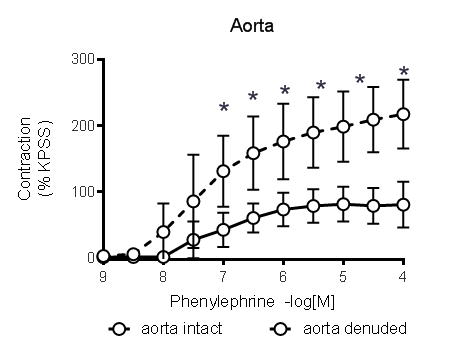

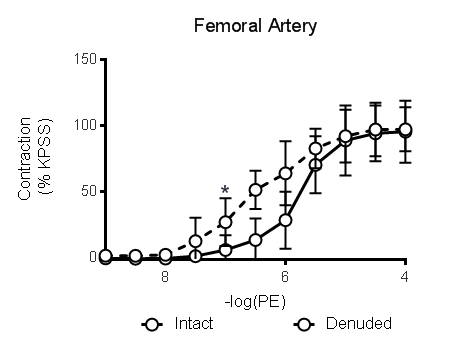

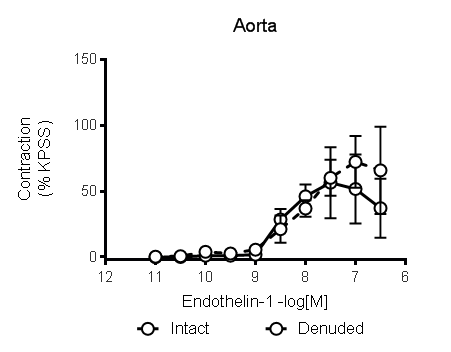

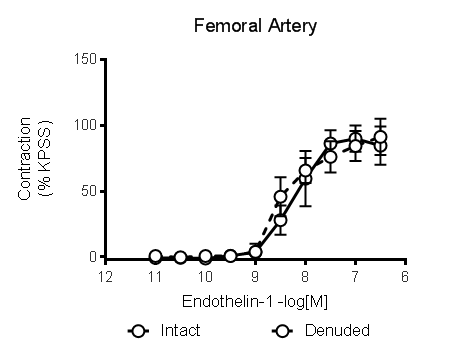


**Supplementary Figure S2**

**Miller *et al.***

(a)

(d)

(b)

(e)

(c)

**Supplementary Figure S2. Impact of endothelial cell removal on functional responses of murine aorta and femoral artery.**

Aorta (a, b, c) and femoral arteries (d, e, f) from adult male C57Bl/6j mice relaxed in response to acetylcholine (ACh) (a, d) and contracted in response to phenylephrine (PE) (b, e) and endothelin-1 (ET-1) (c, f). Removal of the endothelium abolished ACh-mediated relaxation and substantially increased aortic, but not femoral arterial, contraction to PE. It did not, however, alter ET-1 mediated contraction in aorta or in femoral artery. Symbols represent mean+s.e.mean for n= 3 mice. *P<0.05; **P<0.01; ***P<0.001.

(f)


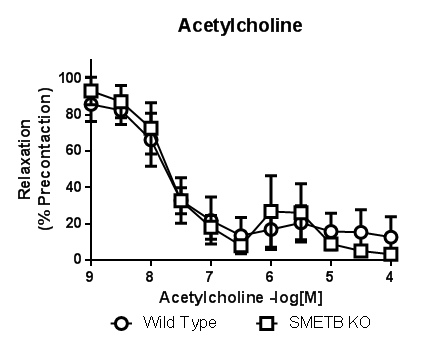

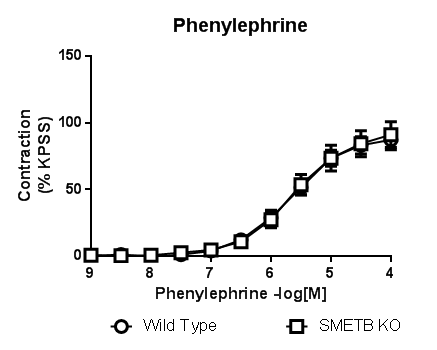

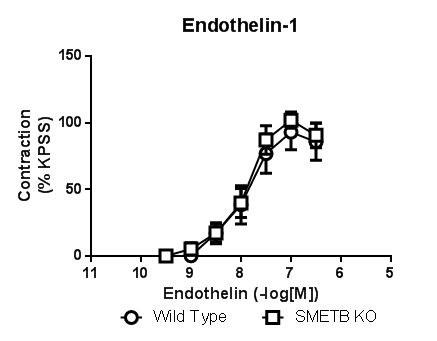


**Supplementary Figure S3**

**Miller *et al.***

(a)

**Supplementary Figure S3. Selective deletion of ET_B_ from the smooth muscle does not alter femoral artery function.**

Concentration-response curves for (a) phenylephrine (PE), (b) endothein-1 (ET-1) and (c) acetylcholine (ACh) were generated in femoral arteries from control (FF--) and SM ET_B_ knockout (FFSm22Cre) mice. Responses to ACh were obtained following sub-maximal contraction with PE. Deletion of ET_B_ had no effect on the responses produced by these agonists. Symbols represent mean+s.e.mean for n=6-7mice.

(c)

(b)


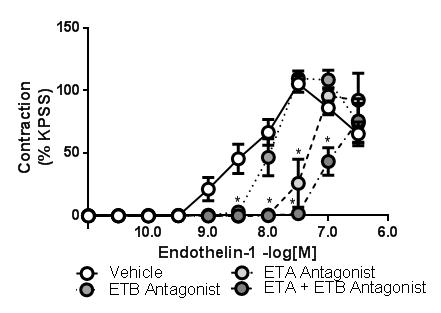


**Supplementary Figure S4**

**Miller *et al***

**Supplementary Figure S4. Endothelin-1-mediated contraction of murine mesenteric arteries is mediated by ET_A_ receptors.**

In murine mesenteric arteries endothelin-1 (ET-1)-mediated contraction was shifted dramatically to the right by incubation with selective ET_A_ (BQ123; 100nM) or mixed ET_A/B_ antagonism, but selective ET_B_ antagonism (A-192621; 100nM) had a much smaller effect. Symbols represent mean+s.e.mean, n=3-7. *P<0.05 compared with Vehicle.


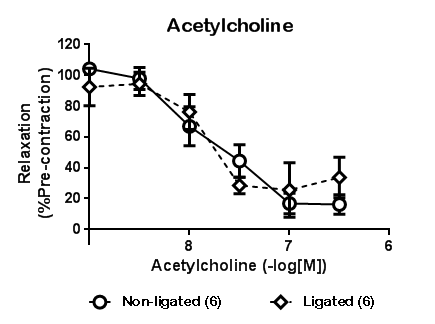

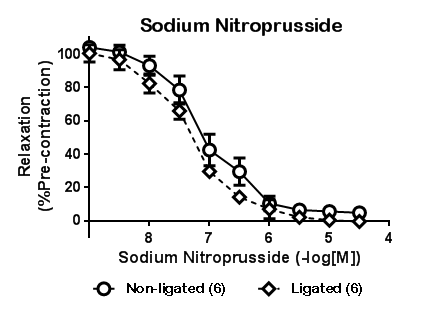

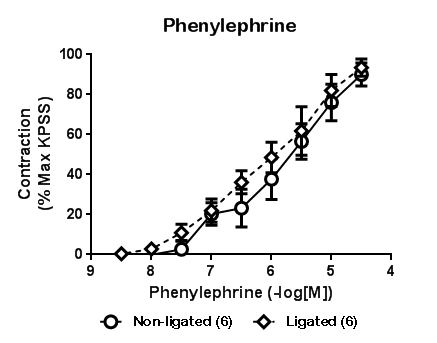


**Supplementary Figure S5. Neointimal lesion formation does not alter functional responses of mouse femoral artery.**

Mouse femoral arteries isolated 28 days after ligation showed unaltered responses to (a) phenylephrine (PE), (b) acetylcholine (ACh) or, (c) sodium nitroprusside (SNP) Data are mean+s.e.mean, n=3-6.

**(a)**

**(c)**

**(b)**

**Miller *et al.***

**Supplementary Figure S5**


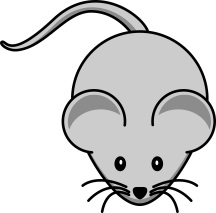

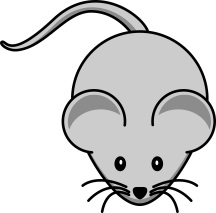


**Supplementary Figure S6. Schematic summary of the effects of smooth muscle cell specific deletion of the Endothelin B receptor (SM ET_B_ KO) in mice.**

**Miller *et al.***

**Supplementary Figure S6**

Neointimal Lesion Formation

**Reduced/Impaired**

**Increased**

ET_B_ Antagonist-induced Hypertension

**Unchanged**

Salt-induced Hypertension

ET_B_ Mediated Contraction

- Trachea (Impaired)

- Mesenteric Vein (Abolished)

- Mesenteric Artery (Abolished)

Heart Rate (Reduced)

Blood Pressure

Plasma [ET-1]

Organ weight

Bodyweight

SMET_B_ deletion
